# Characterization and simulation of conglomerate reservoirs using core data of Triassic Baikouquan Formation, Mahu Depression

**DOI:** 10.1038/s41598-025-09954-4

**Published:** 2025-08-11

**Authors:** Xiangyang Li, Fengxia Li, Zhiwen Huang, Hancheng Ji, Haibo Wang, Liang Chen, Zhonghao Zhang, Xukai Shi

**Affiliations:** 1 SINOPEC Key Laboratory of Shale Oil/Gas Exploration and Production Technology, Beijing, 100083 China; 2 SINOPEC Petroleum Exploration and Production Research Institute, Beijing, 100083 China; 3 State Key Laboratory of Petroleum Resources and Prospecting, Beijing, China; 4 Beijing, College of Geoscience, China University of Petroleum, 18 Fuxue Road, Changping, Beijing, China; 5 Department of Earth and Atmospheric Sciences, Indiana University Bloomington, Bloomington, IN 47405 USA; 6 Tarim Oilfield, Dabei Processing Station, Boda Oil and Gas Production Management Area, CNPC, Aksu, 842305 China; 7 Cup College of Science, China University of Petroleum, 18 Fuxue Road, Changping, BeijingBeijing, 102249 China

**Keywords:** Core samples, Hydraulic fracturing, Reservoir simulation, Conglomerate reservoirs, North slope of Mahu Depression, Junggar Basin, Core processes, Geology, Sedimentology

## Abstract

The 1 Gt oilfield discovery solidified the Mahu oilfield as the world’s largest conglomerate oil region, underscoring the exploration potential of these reservoirs. However, optimizing and selecting the target interval for hydraulic fracturing remains challenging due to the significant heterogeneity of the structure and composition of conglomerate reservoirs. This study addresses key gaps in understanding conglomerate reservoir characteristics and their impact on hydrocarbon production, focusing on the Baikouquan (T_1_*b*) Formation (Fm) on the Mahu Depression’s northern slope. It introduces a new classification to better manage these complexities. In contrast to other classification methods, the proposed approach incorporates key factors influencing hydraulic fracture (HF) propagation, including grain size, cementation, supporting forms, and gravel composition, the latter of which is introduced for the first time. Based on core and test results, the conglomerate reservoirs are categorized into two main groups—fan delta front and fan delta plain conglomerates—and further divided into eight lithofacies types. Fan delta front conglomerates are subdivided into four types: A-1 (tuff, metamorphic, and magmatic rocks-dominated gravel-supported cobble-to-boulder lithofacies), A-2 (tuff and magmatic rocks-dominated matrix-supported pebble-to-cobble lithofacies), A-3 (tuff-dominated matrix-supported granule-to-pebble lithofacies), and A-4 (tuff-dominated gravel-supported granule-to-pebble lithofacies). Fan delta plain conglomerates are further divided into four types: B-1 (tuff and magmatic rocks-dominated gravel-supported granule-to-pebble lithofacies), B-2 (tuff and sedimentary rocks-dominated gravel-supported pebble-to-cobble lithofacies), B-3 (tuff-dominated gravel-supported cobble-to-boulder lithofacies), and B-4 (tuff, magmatic, and sedimentary rocks-dominated matrix-supported pebble-to-cobble lithofacies). The novelty of this classification method lies in its integration of both geological and engineering perspectives, particularly in optimizing hydraulic fracturing strategies. The study evaluates lithofacies from geological factors such as bedding, composition, and poroperm characteristics, as well as engineering considerations like fracturing potential and flow capacity. The results reveal that certain lithofacies types correlate strongly with higher fracturing success, providing insights that can guide more efficient hydraulic fracturing practices. By addressing the challenge of heterogeneity of the structure and composition in conglomerate reservoirs, this study offers a comprehensive framework for selecting optimal target intervals for hydraulic fracturing, which can significantly enhance hydrocarbon exploration and production strategies. This approach is expected to be valuable for similar complex conglomerate reservoirs worldwide.

## Introduction

Conglomerates are coarse-grained sedimentary rocks composed primarily of gravel-sized particles greater than 2 mm in diameter. These rocks typically form in environments with high-energy conditions, such as rivers, beaches, and debris flows. Globally, conglomerate reservoirs have shown immense potential for hydrocarbon exploration, as evidenced by significant discoveries such as the 100 billion m^3^ condensate gas field in Bozhong 19–6 and the 1-billion-ton Mahu oil field in the Mahu Depression^1^. These discoveries underscore the critical importance of understanding conglomerate deposition for improving hydrocarbon reservoir characterization and production.

Conglomerates can be classified according to their genetic origin, composition, and sedimentary features. Common genetic types include alluvial fans, fan deltas, near-shore submarine fans, and lake-bottom fans. Early classifications primarily focused on factors such as lithology, roundness, and grain size, with approaches like Folk’s division of conglomerates into glutenite and breccia, and the classification based on particle size (granules, pebbles, cobbles, and boulders)^2^. More recently, genetic traits such as support form and matrix content have become the foundation for more sophisticated classifications. Researchers like Bluck, Cant and Ethier, and Yu have developed classifications that focus on factors like gravel size, supporting mechanisms, and bedding structure, which are vital for understanding conglomerate distribution and their implications for hydrocarbon exploration^3–5^.

Despite these advancements, traditional conglomerate classification methods have limitations, particularly in the context of unconventional reservoirs. For instance, while these methods effectively differentiate conglomerates based on their depositional environment, they often fail to account for factors critical to the development of unconventional reservoirs, such as hydraulic fracturing. Conglomerate reservoirs, particularly those with low porosity and ultra-low permeability, are increasingly recognized as unconventional resources where hydraulic fracturing is essential for production^6–8^. However, the structure and composition of the conglomerate significantly influence HF propagation, yet many existing classifications overlook this aspect, leaving a gap in understanding how these properties affect fracturing and fluid flow behavior^9^.

This study aims to address this gap by developing a novel classification approach for conglomerate reservoirs, specifically focusing on those within the Baikouquan Fm on the northern slope of the Mahu Depression. This new classification method integrates both geological and engineering considerations, taking into account factors such as grain size, cementation, supporting forms, and gravel composition—especially the latter, which has not been widely incorporated in previous classification systems. By examining thousands of meters of core data, the study seeks to improve our understanding of conglomerate reservoir properties, with a particular focus on optimizing hydraulic fracturing strategies to enhance oil and gas production. This research represents a significant step forward in the characterization of complex conglomerate reservoirs and provides a framework that can be applied to similar unconventional reservoirs worldwide.

## Geological setting

**Fig. 1:**
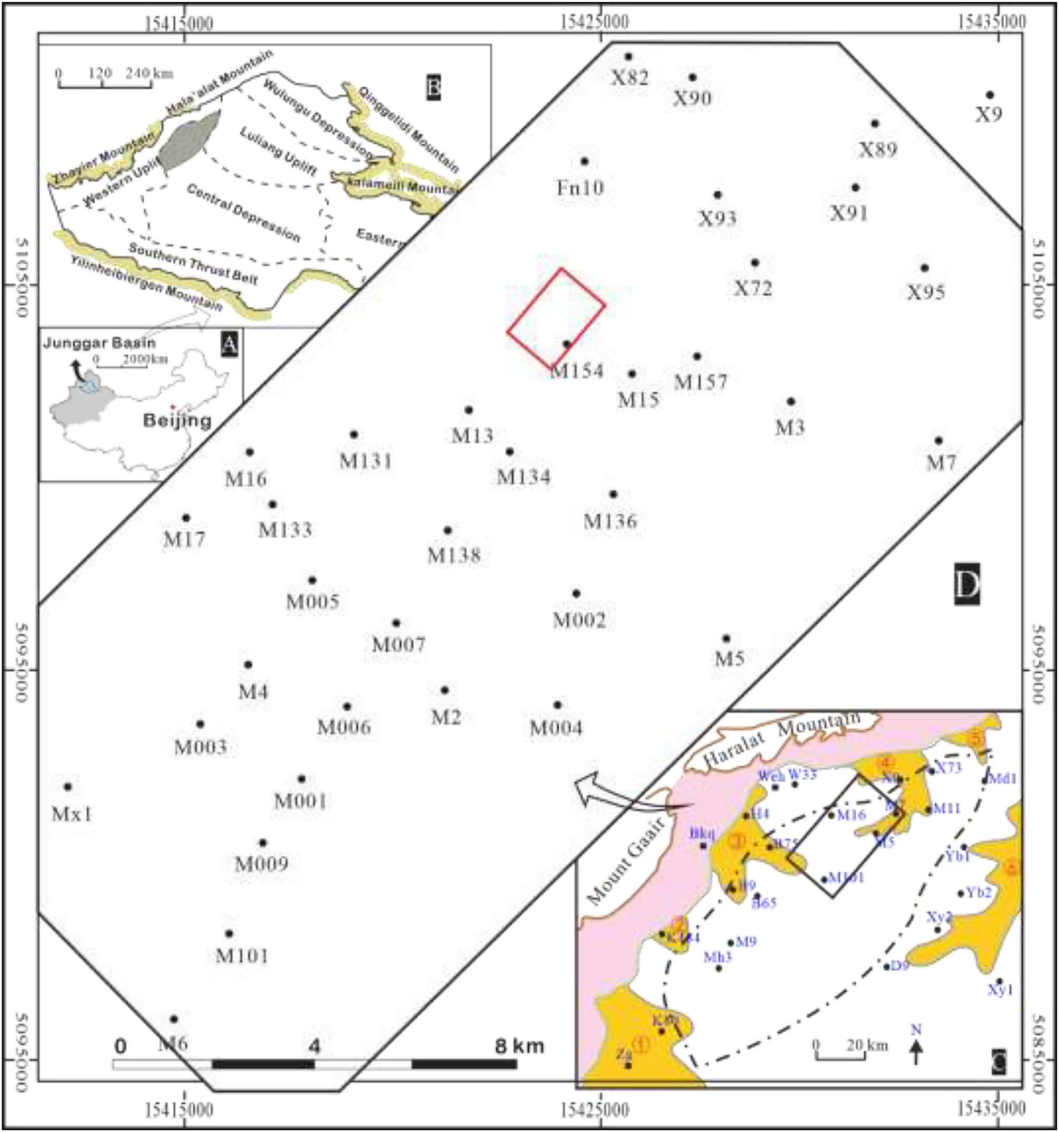
**(A)** Geographical location of Junggar Basin; (**B)** Tectonic unit division of Junggar Basin; (**C)** The locations of the sedimentary systems and study areas of the Triassic Baikouquan Formation in Mahu Depression, ① Zhongguai fan delta, ② Karamay fan delta, ③ Huangyangquan fan delta, ④ Xiazijie fan delta, ⑤ Madong fan delta, ⑥ Xiayan Fan delta; D. An overview of the study area.

One of the most promising petroliferous basins, the Junggar Basin is situated in northern Xinjiang, NW China, and spans an area of roughly 1.34 × 10^5^ km^2^ (Fig. 1A). Its geographic boundaries are the North Tianshan Mountains to the south, the Altai Mountains to the north, and the Zhayier Mountains to the west, as a result, it takes the form of a triangle^10^. According to Wu, Zhao, Yang, and He, the Basin is tectonically located at the junction of the Siberian, Eastern European, and Tarim plates^11–14^. It can be further classified into six first-order zones, which are Wulungu Depression, Luliang Uplift, Western Uplift, Central Depression, Eastern Uplift, and North Tianshan thrust Belt (Fig. 1B).

Situated on the northwest edge of the Junggar Basin, the Mahu Depression is a sub-tectonic unit of the Central Depression of the Junggar Basin. Covering approximately 6.8 × 10^3^ km^2^, it has a virtually NEE striking and is bordered to the west by the foreland uplift belt and to the east by the Luliang Uplift (Fig. 1C). Previous research has indicated that the Permian–Triassic was the primary sedimentary period in the Mahu Depression. The marginal fault belt, which includes the Kebai, Wuxia, and Hongche fault belts, significantly influenced the sedimentary filling of the Mahu Depression^15,16^. Given the intense tectonic activity during the tectonic inversion stage, alluvial-fan, fan-delta, and lake sedimentary facies have primarily evolved in the Mahu Depression^17^. Six fan delta sedimentary systems developed around the Mahu Depression, namely, Xiazijie Fan Delta, Huangyangquan Fan Delta, Karamay Fan Delta, Zhongguai Fan Delta, Yanbei Fan Delta, and Xiyanyan Fan Delta^18^.

Three significant phases may be distinguished in the tectonic history of the Mahu Depression from the Permian to the Triassic: (1) Permian rift from early to middle; (2) tectonic inversion from late Permian to early Triassic; and (3) Depression from middle to late Triassic^19–24^^.^ The depositional period of Triassic Baikouquan Formation in the Mahu Depression represents a sedimentary cycle of retrogradation, which marking the onset of the Depression basin.

**Fig. 2:**
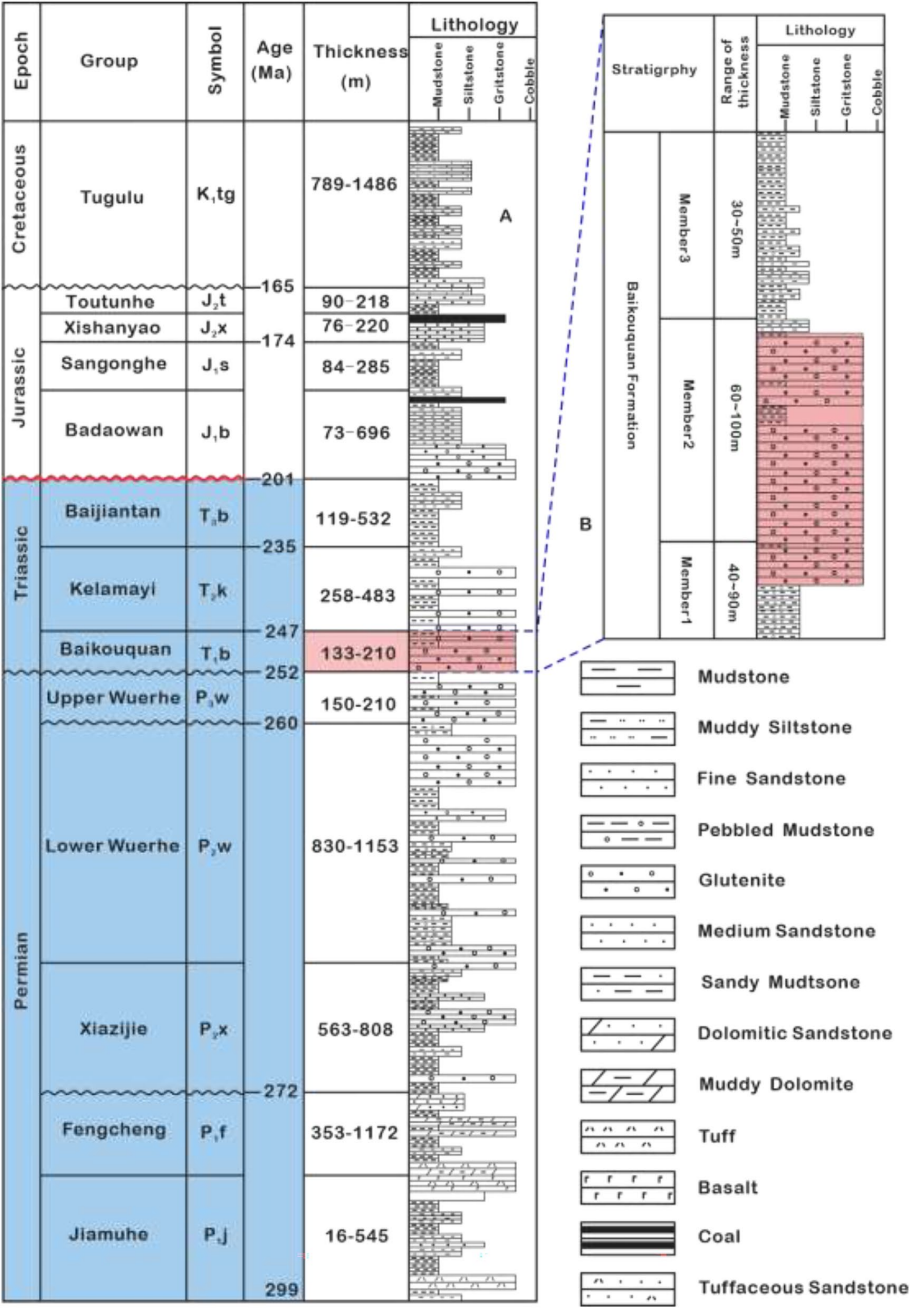
(**A**) Comprehensive stratigraphic column of the Mahu Depression, northwestern Junggar Basin (revised from He)^17^. (**B**) lithologic column and thickness of T_1_*b* in the study area.

The research region is in the northwest of China, in the Junggar Basin, on the northern slope of the Mahu Depression (Fig. 1D). It is distinguished by a monocline that slopes southeastward and a 3°-5° formation dip^25^. With a formation thickness of 130 m-240 m, The target stratum (T_1_*b*) mainly developed conglomerate, sandstone, and mudstone and is subdivided into three groups: (i) a lower group, known as Member 1 (T_1_*b*_*1*_), a thickness of 30 ~ 50 m and gray conglomerate intercalated with brown-gray pebbly mudstone, with massive structure, scour and fill structure, and graded bedding developed ; (ii) a middle group, known as Member 2 (T_1_*b*_*2*_), a thickness of 60–100 m, the lower part developed brown conglomerate, with coarse gravel size and poor sorting, the upper part is dominated by gray-green conglomerate mixed with brown-gray pebbly mudstone; (iii) an upper group, known as Member 3 (T_1_*b*_*3*_), a thickness of 40–90 m, grey-green conglomerate interbedded with mudstone (Fig. 2). The T_1_*b* formation represents a long-term stratigraphic cycle primarily characterized by a regressive fan delta system, with an overall trend of lake transgression. The fan delta front is considered the favorable reservoir zone, with grain-supported granules, glutenite, and pebbly gritstone identified as optimal reservoir facies.

## Methods

### Core sampling and preparation

A total of 1,216.35 m of core samples from 12 wells in the Baikouquan Formation (T_1_*b*) were analyzed. Cores were extracted using standard diamond drilling techniques (Hilti DD 350-Core drill rig) and preserved in sealed containers to prevent oxidation and moisture loss. Prior to analysis, cores were cleaned with deionized water and dried at 60 °C for 48 h in a controlled oven (Memmert UF110). Cores were photographed, slabbed, and logged in 10 cm runs. Samples were then cut into 10 cm segments for macroscopic observation and further subdivided into 2.5 cm × 5 cm subsamples for thin-section preparation and petrophysical testing.

### Macroscopic core observation

Macroscopic analysis followed the workflow outlined by Folk and modified by Liang^2,23^:

1. Color Identification**:** Munsell Rock Color Chart (M50215B) was used under standardized D65 lighting.
2. Gravel Characterization**:**

Size**:** Measured using a gravel template (ASTM D2488-17a) with size classes: granules (2–4 mm), pebbles (4–64 mm), cobbles (64–256 mm), and boulders (> 256 mm).

Sorting**:** Classified as *poor* (> 50% variation), *moderate* (25–50%), or *good* (< 25%) using the Trask sorting coefficient:

$$S_{o} = \sqrt {P_{25} /P_{75} }$$

Roundness**:** Assessed visual chart (angular, subangular, subrounded, rounded).

3. Structural Features**:** Bedding types (massive, graded, cross-bedding) and fracture density (fractures/meter) were recorded using a 10 × hand lens and digital calipers (Mitutoyo 500-196-30). Fracture orientation, length, aperture, and fill, measured with calipers (± 0.01 mm).

### Petrographic analysis

#### Sample preparation

Forty representative samples were prepared following Lakehead University protocols and IUGS guidelines:

1. Cutting and Impregnation: Blocks (2 cm × 2 cm × 1 cm) were cut with a diamond-blade rock saw. Samples were vacuum-impregnated with blue-dyed epoxy to enhance microfracture visibility.
2. Thin Section Preparation:

Grinding and polishing: Mounted samples were ground on a lapping machine using silicon carbide abrasives (down to 600-grit) and polished to a final thickness of 30 µm ± 2 µm, verified with a digital micrometer.

Thin sections were prepared using a Buehler PetroThin system.

#### Microscopy and mineral identification

1. Thin sections were analyzed using two polarizing microscopes:

Leica DM2700P with 5 × –40 × objectives.

Nikon LABOPHOT-2 under plane-polarized (PPL) and cross-polarized light (XPL) at 25 × –400 × magnification.

1. Thin sections were analyzed using two polarizing microscopes:
2. Mineral identification utilized:

Interference colors and extinction angles under XPL.

Michel-Lévy birefringence charts for birefringence contrast optimization (adjusting condenser iris and retardation plate).

#### Compositional and textural analysis

1. Gravel Composition:

Point-counted 300 grains per slide using JMicroVision 1.3.4 software.

1. Thin sections were analyzed using two polarizing microscopes:
2. Cementation Types:
  Differentiated calcite, ferric calcite, and clay minerals via staining: Dickson’s solution for carbonates and potassium ferricyanide for iron detection.
3. Image Analysis:

Photomicrographs captured with a Nikon DS-5Mc camera.

Mineral abundances quantified using ImageJ: Color-threshold segmentation applied to 10 non-overlapping fields per sample, yielding ± 2% uncertainty.

### Porosity and permeability measurements

#### Porosity

The core plug was saw-cut, sanded smooth, cleaned in ultrasonic bath with methanol for 10 min, then dried at 60 °C under vacuum (10⁻^2^ bar) for 24 h. It (2.5 cm diameter) was analyzed using a AP-608 Automated Porosimeter (Core Laboratories) at 500 psi confining pressure. Porosity (*ϕ*) was calculated as:

$$\phi \, = \,V_{{{\text{pore}}}} /V_{{{\text{bulk}}}} \, \times \,{1}00\%$$

where* V*_pore_ was derived from Boyle’s law and* V*_bulk_ caliper measurements.

Validation: Repeatability tested with three measurements per sample; standard deviation < 0.5%.

#### Permeability

Permeability was measured on the same plugs using a steady-state nitrogen permeameter (OFITE BLP-530) at room temperature. The experiment utilized a core holder with Viton® O-rings to seal plugs under 500 psi confining pressure to prevent gas bypass. Nitrogen was flowed at 50 psi upstream pressure (downstream at atmospheric pressure), with stabilized gas flow rates measured via a calibrated soap-bubble flowmeter after 10 min. Flow and pressure differential (ΔP) data were recorded every 30 s over 5 min to average fluctuations. Permeability (K*K*) was calculated via Darcy’s law:

$$K = \frac{Q*U*l}{{A*\Delta P}}$$

where *Q* = flow rate (cm^3^/s), *μ* = gas viscosity (0.0178 cP for N₂), *L* = core length (cm), *A* = cross-sectional area (cm^2^), and Δ*P* = pressure differential (atm).

Quality Control: Confined to samples with < 5% clay content to avoid fines migration artifacts.

### Grain size analysis

To determine gravel size distribution, we employed the ASTM D6913-04 method:

1. Sample crushing: 200 g core fragments were crushed in a jaw crusher to < 25 mm, cleaned of fines by rinsing and oven-drying at 105 °C for 12 h.
2. Sieving: A nested stack of ASTM standard sieves (25 mm, 12.5 mm, 6.3 mm, 2 mm, 1 mm, 0.5 mm) was arranged; samples were sieved for 15 min in a mechanical shaker (200 rpm).
3. Mass measurement: Retained fractions were weighed on an analytical balance (± 0.01 g).
4. Data processing: Mass percentages were calculated for each size class, and cumulative curves plotted to derive D₁₀, D₅₀, D₉₀ values using Grading software. Repeats showed < 3% variation.

### Statistical analysis

All data were processed in SPSS 27.0. Porosity–permeability correlations used Pearson’s *r*; lithofacies differences were tested via ANOVA (*p* < 0.05).

## Results

### Why this categorization

The T_1_*b* Formation in the North Slope of the Mahu Depression is characterized by proximal coarse-grained retreating fan-delta deposition systems, significantly influenced by the depositional systems originating from the Xiazijie fan in the northeast and the Huangyangquan fan in the southwest. As a result, the conglomerates in the region are polymictic, with the dominant component being volcaniclastic material, followed by magmatic, sedimentary, and metamorphic rocks^26,27^. Tuff, primarily composed of pyroclastic rock, constitutes about 50% of the gravel component, while granite, andesite, and rhyolite represent the dominant magmatic rocks, and quartzite and metasandstone are the primary metamorphic constituents. Sedimentary rocks, including siliceous rock and mudstone, make up the remaining portion^28^.

Moreover, the sandstone component of the conglomerates closely matches the gravel component, with tuff making up approximately 35% of the total sandstone, alongside trace amounts of sedimentary, magmatic, and metamorphic rocks. Detailed core observations and microscopic thin section analysis have revealed significant variations in the composition, gravel size, support type, and cementation of the conglomerates, indicating considerable spatial heterogeneity. For example, in well M006, located within the study area, core intervals demonstrate marked variations in gravel composition. While tuff predominates in barrels 1–6, mudstone becomes the dominant component in barrels 11–14. These variations have a direct impact on the rock’s mechanical properties, which in turn affect fracture propagation. It has been established that changes in gravel composition led to significant variations in rock mechanical properties, which influence the geometry and extension of fractures^29,30^.

Traditional conglomerate classification systems, which are primarily based on geological factors, largely overlook the impact of reservoir simulation, particularly hydraulic fracturing. The structure, composition, and cementation of conglomerates have a profound effect on the propagation of HFs. Given the complexity of the conglomerates in the T_1_*b* Formation, a new classification system that considers both geological and engineering factors is needed to improve the accuracy of reservoir characterization and optimize hydraulic fracturing. The following sections outline the new categorization system proposed in this study, which integrates these factors to provide a more comprehensive understanding of the conglomerate reservoirs.

### The conglomerate classification

#### Initial classification based on genesis

Conglomerates and pebbly sandstones, which form the primary deposits of the Baikouquan Formation in the North Slope of the Mahu Depression, exhibit significant deposition thickness, diverse structure, and component variation^31^. To investigate the spatial heterogeneity and differential distribution of these conglomerates, the study utilized a detailed description of a 1216.35 m core section. Based on previous research and thorough observation of the core, the conglomerates of the T_1_*b* Formation were initially divided into fan delta front and fan delta plain conglomerates based on their genesis.

**Fig. 3:**
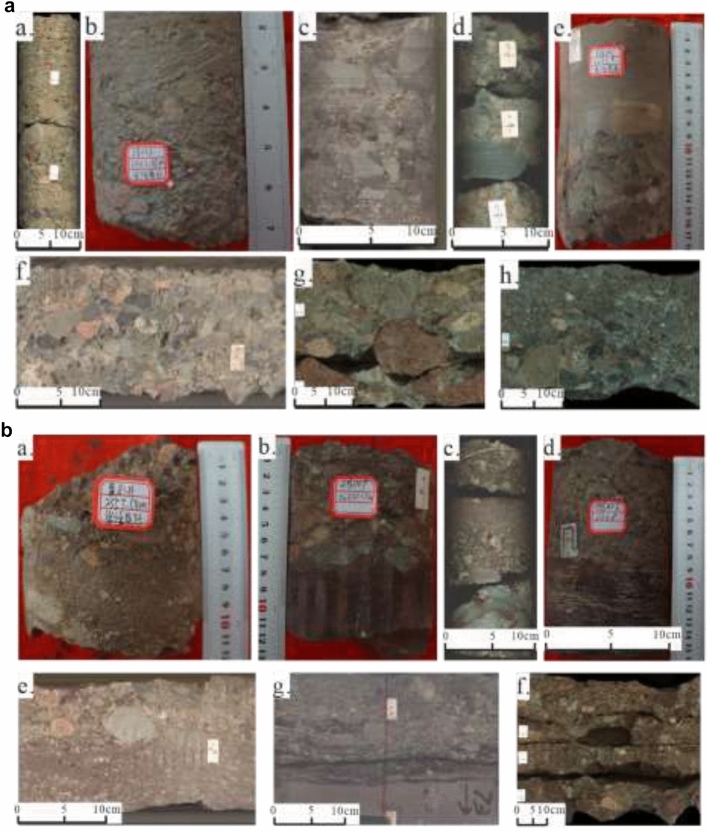
**(a)** Core data shows the lithofacies of the fan delta front conglomerate in the Baikouquan Formation (T_1_*b)*, Northern Slope, Mahu Depression. **a**. M13, 3110 m, T_1_*b*_3_, greyish-green sandstone-supported pebble, the gravel is weakly oriented and mainly cemented by calcite; (**b)** M002, 3442.35 m, T_1_*b*_3_, gray sandstone-supported granule-to-pebble, medium sorted with inverse grading; (**c)** M6, 3873.1 m, T_1_*b*_2_, gray gravel-supported pebble, poorly sorted with massive structure developed; (**d)** M2, 3418.21 m, T_1_*b*_2_, motley matrix-supported pebble-to-cobble, extremely poorly sorted with massive structure; (**e)** M001, 3472.9 m, T_1_*b*_2_, gravel-bearing medium sandstone at the top and gray-green gravel-supported pebble-to-cobble at the bottom; the conglomerates are generally poorly sorted with massive structure developed, a lithologic discontinuity surface exists between the conglomerate and sandstone; (**f)** M003, 3486.8 m, T_1_*b*_2_, jade-green gravel-supported pebble-to-cobble, poorly sorted with massive structure developed; (**g**) M132, 3274.31 m, T_1_*b*_3_, gray matrix-supported cobble-to-boulder, extremely poorly sorted with rounding of mainly sub-rounded, and developed massive structure and normal grading; h. X89, 2503.12 m, T_1_*b*_2_, gray-green matrix-supported pebble-to-cobble, moderately sorted and wide rounding of sub-angular to sub-rounded with normal grading and gravel weakly oriented arrangement developed.

The distinction between fan delta front and fan delta plain conglomerates lies in several geological and sedimentary features. Fan delta front conglomerates are typically associated with a higher-energy environment, marked by better sorting, sub-rounded to rounded gravel, and the presence of more defined bedding structures, such as rhythmic bedding and imbricated structures. In contrast, fan delta plain conglomerates are deposited in lower-energy environments, exhibiting poorer sorting, a wider range of gravel sizes, and more matrix-supported gravel with weakly developed structures. The fan delta plain conglomerates are primarily characterized by oxidized colors (dark brown, taupe, purple, etc.), poor sorting, and subangular to sub-rounded gravel (Fig. 3a), whereas the fan delta front conglomerates have reduced colors (green gray, brown gray, dark gray, etc.), better sorting, and are predominantly matrix-supported with evidence of debris flow and traction current deposits (Fig. 3b). This classification scheme is based on the principle that these two depositional environments exhibit distinct characteristics that influence both reservoir quality and the potential for hydraulic fracturing.

#### Subsequent classification based on geological and engineering considerations

To account for both geological characteristics and engineering factors (particularly the impact of hydraulic fracturing), the conglomerates were further subdivided into four types based on a combination of four key factors: gravel size, supporting form, cementation strength, and gravel component composition. These factors were chosen because they directly influence both the geological properties of the reservoir and its mechanical response to hydraulic fracturing.

Figure 3 b Core data shows the lithofacies of the fan delta plain conglomerate of Baikouquan Formation (T_1_*b)*, Northern Slope, Mahu Depression. a. X81, 2557.54 m, T_1_*b*_2_, motley gravel-bearing coarse sandstone, moderately sorted; b. M009, 3634.15 m, T_1_*b*_1_, the upper part grows maroon mudstone-supported pebble, moderately sorted, wide rounding of sub-angular to sub-rounded, and with a massive structure developed, the lower part grows maroon mudstone, a lithologic discontinuity surface exists between the upper conglomerate and the lower mudstone; c. M2, 3461.41 m, T_1_*b*_1_, maroon matrix-supported pebble-to-cobble, extremely poorly sorted with rounding of sub-angular, and with massive structure and normal grading developed; d. M006, 3458 m, T_1_*b*_1_, brownish-grey matrix-supported granule at the top, the gravels have a moderate to poor sorting and a wide rounding of sub-angular to sub-rounded with gravel weakly oriented arrangement growth, the lower part is tan siltstone with lithologic discontinuity surface in the middle part; e. M003, 3547.08 m, T_1_*b*_1_, brown matrix-supported pebble-to-cobble, poorly sorted and wide rounding of sub-angular to sub-rounded with massive structure and normal grading growth; f. M001, 3487.34 m, T_1_*b*_2_, the upper part grows matrix-supported granule-to-pebble, moderately sorted and rounding of sub-angular with massive structure and gravel weakly oriented arrangement developed, the middle part grows brown gravel-bearing mudstone and a lithologic discontinuity surface developed in the middle of them, the lower part grows brown gravel-bearing fine sandstone; g. M132, 3475.02 m, T_1_*b*_3_, brown matrix-supported pebble, moderately sorted, wide rounding of sub-angular to sub-rounded with massive structure developed.

**Fig. 4:**
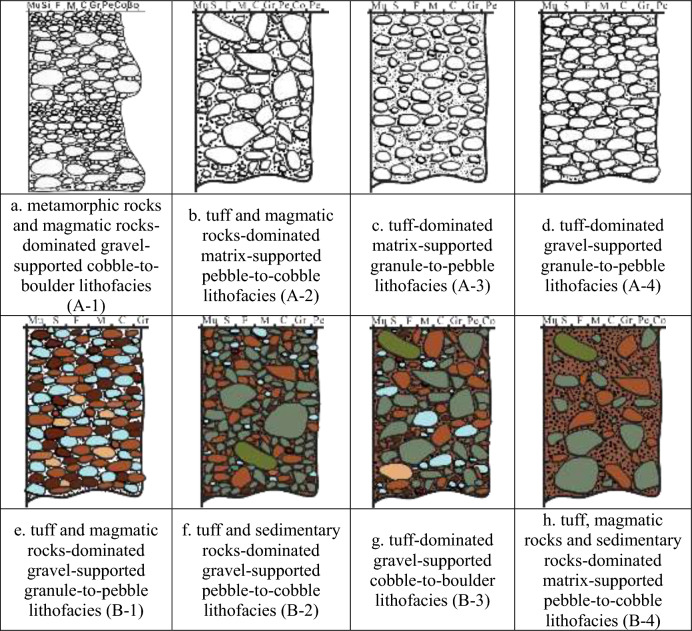
Lithofacies types of fan delta conglomerate in Baikouquan Formation, North Slope of Mahu Depression.

The classification of the T_1_*b* conglomerates into fan delta front and fan delta plain conglomerates was further refined into four subclasses each. The four types for fan delta front conglomerates are: A-1: Tuff, metamorphic, and magmatic rock-dominated gravel-supported cobble-to-boulder lithofacies (Fig. 4a); A-2: Tuff and magmatic rock-dominated matrix-supported pebble-to-cobble lithofacies (Fig. 4b); A-3: Tuff-dominated matrix-supported granule-to-pebble lithofacies (Fig. 4c); A-4: Tuff-dominated gravel-supported granule-to-pebble lithofacies (Fig. 4d). Similarly, the fan delta plain conglomerates were divided into the following four types: B-1: Tuff and magmatic rock-dominated gravel-supported granule-to-pebble lithofacies (Fig. 4e); B-2: Tuff and sedimentary rock-dominated gravel-supported pebble-to-cobble lithofacies (Fig. 4f); B-3: Tuff-dominated gravel-supported cobble-to-boulder lithofacies (Fig. 4g); B-4: Tuff, magmatic rock, and sedimentary rock-dominated matrix-supported pebble-to-cobble lithofacies (Fig. 4h).

This classification method incorporates factors such as cementation strength and gravel composition, which are critical for understanding the mechanical behavior of the reservoir and predicting its response to hydraulic fracturing. By combining these factors, the classification system provides a more accurate and holistic understanding of the T1b conglomerate reservoirs.

### The conglomerate features

#### Fan delta front conglomerates

1. Class A-1 is metamorphic rocks and magmatic rocks-dominated gravel-supported cobble-to-boulder lithofacies (Tables 1, 2, 3 and 4). Thick, massive rock, primarily grey and greenish grey in hue, with a maximum thickness of 3 m, forms A-1. The lithofacies, interbedded with thin pebbled sandstone or dark mudstone with a thickness of 10 cm to 30 cm, develops normal rhythms, graded beddings, weakly gravel orientations, and additional stratifications. Between pebbly sandstone or mudstone and class A-1 is an erosional surface. The bottom gravels with larger sizes that go beyond boulders are matrix-supported, while towards the top of the class, the gravel gets smaller and becomes gravel-supported. The overall rounding is sub-angular to sub-rounded and sub-rounded, with the worst sorting at the bottom improving progressively toward the top.

**Table 1:** Overall characteristics of the fan delta front conglomerate of Baikouquan Formation (T1b), Northern Slope of Mahu Depression.

Litho	Well-core	Rock type	Matrix	Cement	Pyr	Mag	Met	Sed
A-1	X82-4	Co	Low	High	Tuff	F., A., G	Me	–
	X82-5	Co	Low	Low	Tuff	F., A., R., G	Me	–
	X89-6	Co	Low	Low	Tuff	A	Me., Q	–
	X89-7	Co	Low	High	Tuff	A	Me., Q	–
	X90-3	Co	Low	High	Tuff	F	–	–
A-2	M132-1/2/3	Co	High	Low	Tuff	F., A	–	Mu
	X94-3	Co	High	Low	Tuff	F., A., R	Me., Q	Mu
	M002-7	Pe.-Co	High	Low	Tuff	F., A., R., G	Me., Q	Mu
	M002-6	Pe.-Co	High	Low	Tuff	F., A., R., G	–	S
	M2-27	Pe.-Co	High	Low	Tuff	F., A., R., G	–	S., Mu
	M133-2	Pe.-Co	High	Low	Tuff	F., A	–	–
	M133-3	Gr.-Pe	High	Low	Tuff	F., A	Me., Q	Mu
A-3	M13-1	Ps	High	High	Tuff	–	–	–
	M003-3	Ps	High	High	Tuff	–	–	S
	M005-2	Gr.-Pe	Low	Low	Tuff	G	–	–
	M15-3	Gr.-Pe	High	High	Tuff	F., A., R	Me., Q	–
	M16-1	Ps	High	Low	Tuff	F., A., G	Me	Mu
	M7-5	Gr.-Pe	High	Low	Tuff	A., R	–	–
	M006-3	Gr.-Pe	High	Low	Tuff	A., G	Me	Mu
	M006-4	Gr.-Pe	High	Low	Tuff	A., G	Me	Mu
A-4	M003-4/5–1	Pe	Low	Low	Tuff	G	–	–
	M003-4/5–2	Gr.-Pe	Low	Low	Tuff	G	–	Mu
	M005-6	Gr.-Pe	Low	Low	Tuff	–	–	Mu
	M6-4/5	Gr.-Pe	High	Low	Tuff	G	–	Mu
	M101-2	Gr.-Pe	Low	Low	Tuff	G	–	Mu
	M005-4	Gr.-Pe	Low	Low	Tuff	R., A	–	Mu
	M131-2	Pe	Low	Low	Tuff	F., A., R., G	–	S
	M001-5	Gr.-Pe	Low	Low	Tuff	R., G	Q	–
	M001-6	Pe	Low	Low	Tuff	R., A	–	–
	M002-5	Gr	High	Low	Tuff	F., A., R., G	–	S
	M11-2	Gr	Low	Low	Tuff	A., G	–	S

Where Co., Pe., Gr., Ps. represent Cobble, Pebble, Granule, Pebbly sandstone, respectively; F. A., R., G. represent Felsite, Andesite, Rhyolite, Granite, respectively; Me., Q. represent Metasandstone, Quartzite, respectively; Mu. and S. represent Mudstone and Silicalite, respectively.

**Table 2:** Structure feature of the fan delta front conglomerate of Baikouquan Formation (T1*b*), Northern Slope of Mahu Depression.

Litho	Well-core	Supporting	Sorting	Structure	Petrographic color	Rounding
A-1	X82-4	GS	Bad	Mas	Green grey	Sa. to Sr., Sr
	X82-5	GS	Bad	Mas	Green grey	Sa. to Sr., Sr
	X89-6	GS	Poor	Mas	Celadon	Sa. to Sr
	X89-7	GS	Poor	Mas., Gwo	Celadon	Sa. to Sr
	X90-3	GS	Poor	Mas., Nr	Grey	Sr
A-2	M132-1/2/3	GS	Poor	Mas., Nr	Grey	Sr
	X94-3	Ms	Bad	Mas., Nr., Gwo	Green grey, Dark grey	R. to Sr
	M002-7	Ms	Worse	Mas., Gwo., Rr	Gray	Sr
	M002-6	Ms	Poor	Nr., Mas., Gwo	Mottle, Green grey	Sr., Sa. to Sr
	M2-27	Ms	Poor	Mas	Gray, field grey	Sa., Sr
	M133-2	Ms	Bad	Mas., Nr., Rr	Mottle	R
	M133-3	Ms	Worse	Mas., Gwo., Rr	Gray	Sa.-Sr
A-3	M13-1	Ss	Bad	Gwo	Green grey, Grey	Sr
	M003-3	Ss	Bad	Mas., Nr., Gwo	Gray, Taupe	Sa. to Sr
	M005-2	Ss	Bad	Rr., Nr., Gwo	Grey	Sa. to Sr
	M15-3	Ss	Worse	Mas	Grey	Sr
	M16-1	Ss	Worse	Mas	Gray, Taupe	R. to Sr
	M7-5	Ss	Worse	Mas	Celadon	Sr
	M006-3	Ss	Bad	Mas., Nr	Gray, Green, Celadon	Sa., Sa. to Sr
	M006-4	Ss	Bad	Mas., Nr	Taupe, Brown–red	Sa., Sa. to Sr
A-4	M003-4/5–1	GS	Bad	Mas., Rr., Gwo	Green grey	Sa
	M003-4/5–2	GS	Worse	Mas., Rr., Gwo	Green grey	Sa., Sa. to Sr
	M005-6	GS	Medium	Mas	Grey, Dark grey	Sa., Sa. to Sr
	M6-4/5	GS	Medium	Mas., Nr., Gwo	Gray, Dark grey	Sa
	M101-2	GS	Medium	Mas., Nr., Gwo	Grey, Dark grey	Sa
	M005-4	GS	Worse	Mas., Nr	Celadon	Sa
	M131-2	GS	Worse	Mas., Nr	Celadon	Sa
	M001-5	GS	Worse	Mas., Nr	Celadon	Sa. to Sr., Sr
	M001-6	GS	Bad	Mas., Nr., Gb	Celadon, Green grey	Sa
	M002-5	GS	Worse	Mas., Nr	Celadon	Sa. to Sr., Sr
	M11-2	GS	Bad	Mas., Nr., Gb	Celadon, Green grey	Sa

Where GS., Ms., Ss. represent Gravel-Supported, Mudstone-Supported, Sandstone-Supported, respectively; Ms. Gwo., Nr., Rr., Gb. represent Massive structure, Gravel weakly-oriented, Normal rhythm, Reverse rhythm, Graded bedding, respectively; Sa., Sr., R. represent Sub-angular, Sub-rounded, Rounded, respectively.

**Table 3:** Component analysis of the fan delta front conglomerate of Baikouquan Formation (T_1_*b*), Northern Slope of Mahu Depression.

Litho	Well-core	All	Tuff/%	F./%	R.%	A./%	Me./%	G./%	S./%	Q./%	Mu./%
A-1	X82-4	75.63	26.75	21.5	3.75	8.13	8.5	4.5	–	2.5	–
	X82-5	83.86	24.83	1/.67	11.17	12	9.17	5.67	–	9.69	/.67
	X89-6	81.5	29	–	–	11.67	2/.83	3.33	–	16.17	/.5
	X89-7	78.5	28.13	–	–	1/	19.13	1.5	–	19.75	–
	X9/-3	8/	26	6	–	14	34	–	–	–	–
A-2	M132-1/2/3	62.33	33.33	6.33	–	6.67	/	1.67	–	–	14.33
	X94-3	67.75	17.5	7	3.25	11.75	21.75	–	–	2.5	4
	M//2–7	58.5	34	3	1.5	4	–	5.5	/.5	–	1/
	M//2–6	62	27.17	3.83	2.67	5.5	2.33	7.33	/.33	2.17	1/.67
	M2-27	57.7	23.2	4.2	6.7	9.5	1.1	1.7	1.9	/.4	9
	M133-2	58	28	15	–	5	–	–	–	–	1/
	M133-3	51.5	13	12.5	2.5	4.5	2.5	2.5	/	1	13
A-3	M13-1	13.67	1/.67	1	–	2	–	–	–	–	–
	M//3–3	43.33	35.67	–	–	1.67	–	–	6	–	–
	M//5–2	51	27	–	–	–	–	24	–	–	–
	M15-3	4/	3/	–	4	3	2	–	–	–	1
	M16-1	5/	15.5	11	–	5	6.5	5	–	3	4
	M7-5	51.5	14.5	–	8.5	8.5	–	6	7	2	5
	M//6–3	45.5	26.69	–	–	2.69	6.56	2./6	–	/.25	7.25
	M//6–4	62.33	29.45	–	–	6.12	11.51	8.25	–	–	7
A-4	M//3–4/5–1	87	67	–	–	–	–	2/	–	–	–
	M//3–4/5–2	73.67	58.33	–	–	2.67	–	4.33	2	–	6.33
	M//5–6	77	64.67	–	–	2.67	–	–	–	–	9.67
	M6-4/5	58.43	38	–	–	–	1.86	6.71	1.14	–	1/.71
	M1/1–2	81	47.67	–	–	–	–	12.33	–	–	21
	M//5–4	81	63.4	–	6	–	–	4.6	–	–	7
	M131-2	84.2	36.2	1/.8	17.4	6.6	–	4.6	8.6	–	–
	M//1–5	69.67	61.33	–	2.67	–	–	1.67	/	4	–
	M//1–6	63	31.33	–	16.33	12.67	–	–	–	2.67	–
	M//2–5	85.25	3/	12.75	14	7.5	–	12.5	8.5	/	–
	M11-2	66.5	27.5	–	–	14	–	19	6	/	–

**Table 4:** Fill characteristics of the fan delta front conglomerate of Baikouquan Formation (T_1_*b*), Northern Slope of Mahu Depression.

Litho	Well-core	Matrix/%	Matrix component	Cement/%	Cement component
A-1	X82-4	0.52	Mud	4.75	Analcite, Calcite
	X82-5	0.83	Mud, Kaolinite	4.31	Calcite
	X89-6	1.5	Mud	2.33	Calcite
	X89-7	2.13	Mud	3.25	Calcite
	X90-3	0.87	Mud	5.4	Analcite, Calcite
A-2	M132-1/2/3	7	Chlorited-mud, Mud, Kaolinite	–	Ferriferous calcite
	X94-3	7	Chlorited-mud, Mud, Kaolinite	–	Ferriferous calcite
	M002-7	7	Mud, Kaolinite, Chlorite	–	Ferriferous calcite
	M002-6	7.83	Mud, Kaolinite, Chlorite	0.17	Calcite
	M2-27	8.6	Chlorited-mud, Kaolinite	0.7	Ferriferous calcite
	M133-2	14	Kaolinite	–	–
	M133-3	5.5	Mud, Kaolinite	–	–
A-3	M13-1	5.33	Mud, Kaolinite	0	Calcite
	M003-3	3	Oil stains mud, Kaolinite, Mud	3.33	Calcite, Ferriferous calcite
	M005-2	2	Mud	7	Ferriferous calcite
	M15-3	3	Chlorited-mud	8	Calcite
	M16-1	2	Mud	8	Calcite
	M7-5	1.5	Mud, Kaolinite	8	Calcite
	M006-3	2.3	Mud, Kaolinite, Chlorite, Hydrobiotite	5.63	Calcite
	M006-4	2.66	Hydromica, Mud	2.51	Calcite
A-4	M003-4/5–1	3	Chlorite, Mud	–	Calcite
	M003-4/5–2	3	Mud, Kaolinite, Chlorite, Hydromica	–	Calcite
	M005-6	2	Mud, Kaolinite	1.33	Ferriferous calcite, Calcite
	M6-4/5	5.14	Hydrobiotite, Kaolinite, Mud	–	–
	M101-2	5.14	Hydrobiotite, Kaolinite, Mud	–	–
	M005-4	3.6	Mud, Chlorite, Kaolinite	0.2	Calcite
	M131-2	2	Kaolinite, Mud	–	–
	M001-5	3.67	Kaolinite, Mud, Chlorite	–	Calcite
	M001-6	3	Kaolinite, Hydromica, Chlorite	–	Calcite
	M002-5	4.5	Kaolinite, Chlorite, Mud	0.25	Calcite
	M11-2	4.5	Kaolinite, Chlorite, Mud	0.25	Calcite

**Fig. 5:**
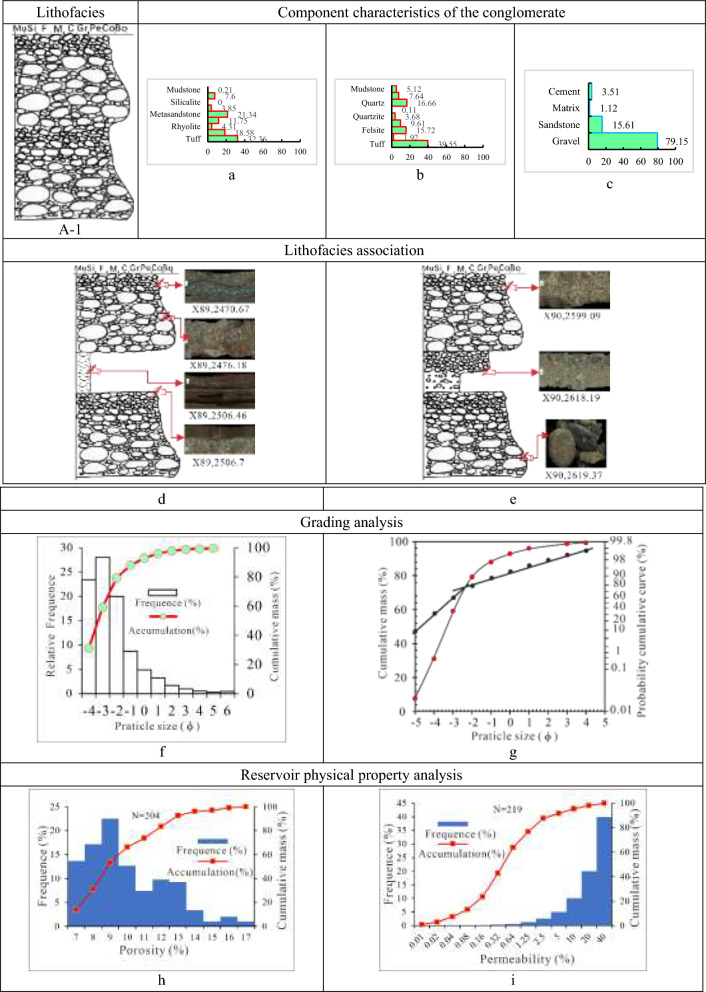
Parameter characteristics of the delta front conglomerate of class A-1 in the Baikouquan Formation (T_1_*b*), Northern Slope of Mahu Depression. (**a)** Gravel component (%), (**b)** Sandstone component (%); (**c**) Conglomerate composition (%); (**d**) The conglomerate is interlayered with thin brown mudstone, with weakly gravel orientations developed in the top; (**e**) The conglomerate was cemented with calcite and developed massive structure, with boulder growing in the bottom; (**f)** Grain distribution histogram and cumulative grading curve; (**g)** Cumulative probability curve; (**h)** Histogram of porosity distribution; (**i)** Permeability distribution histogram.

With an average gravel content of 69.9%, the lithofacies A-1 is mostly composed of volcaniclastic rocks (tuff), metamorphic rocks (quartzite and metasandstone), and magmatic rocks (felsite and andesite), accounting for 32.4%, 38.5%, and 29.0%, respectively. The cement is calcite, with a high average percentage of 6.0%, while the filler matrix is mud, with a low average content of 2.0%. The reservoir of the lithofacies is poorly analyzed, with a permeability of (0.2–5) × 10^–3^ µm^2^ and a porosity of 7%-14%. The deposit type of this lithofacies is associated with the fan delta front main channel, with a grain distribution curve of unimodal mode distribution and a cumulative curve of two parts (rolling and jumping) that is dominated by rolling overall (Fig. 5).

(2) Class A-2 is tuff and magmatic rocks-dominated matrix-supported pebble-to-cobble lithofacies (Tables 1, 2, 3 and 4). Thick, massive rock, primarily grey, brown grey, and dust grey in hue, with a maximum thickness of 2 m to 3 m, form A-2. The lithofacies, interbedded with thin pebbled sandstone or dark mudstone with a thickness of 10 cm to 30 cm, develops normal rhythms, massive structures, gravel orientations, and additional stratifications. And between sandstone or mudstone and conglomerate is a distinct erosional surface. The conglomerate in this lithofacies, mainly supported by matrix, has a medium to coarse gravel grade, poor sorting, and moderately well rounding of sub-angular to sub-rounded.

**Fig. 6:**
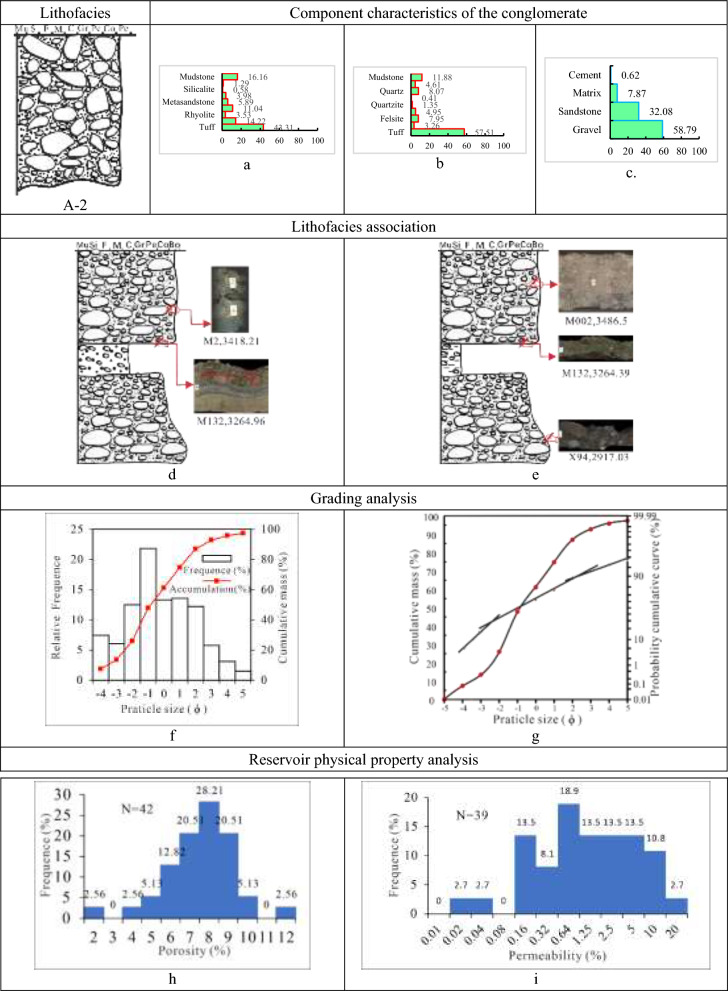
Parameter characteristics of the delta front conglomerate of class A-2 in the Baikouquan Formation (T_1_*b*), Northern Slope of Mahu Depression. (**a)** Gravel component (%), (**b)** Sandstone component (%); (**c)** Conglomerate composition (%); (**d)** The conglomerate is interlayered with thin pebbly sandstone, with weakly gravel orientations developed; (**e)** The conglomerate is interlayered with thin brown mudstone and develops massive structure; (**f)** Grain distribution histogram and cumulative grading curve; (**g)** Cumulative probability curve; (**h)** Histogram of porosity distribution; (**i)** Permeability distribution histogram.

With an average gravel content of 58.8%, the lithofacies A-2 is dominated by volcaniclastic rocks (tuff) and magmatic rocks (felsite and andesite), accounting for 43.3% and 32.8%, respectively, followed by sedimentary rock (mudstone), to a lesser extent, accounting for 16.7%. The sedimentary rock content in the gravel composition increases while the metamorphic rock content decreases in comparison to Class A-1. The cement is calcite and ferriferous calcite, with a low average percentage of 0.6%, while the filler matrix is mud, kaolinite, and chlorite, with a high average content of 7.8%. The A-2 lithofacies porosity histogram displays an unimodal mode with a value of 6%-9%. In contrast, the permeability histogram shows a low unimodal mode with a wide range of variation, with a value of (0.2–10) × 10^–3^ µm^2^. By contrast, the physical properties of Class A-2 conglomerates are inferior to Class A-1. This lithofacies’ deposit type links to the fan delta front branch channel, of which the cumulative curve has a low slope and no evident node, and the grain size histogram displays a low unimodal mode distribution, indicating that the gravel has a wide grain distribution and poor sorting (Fig. 6).

(3) Class A-3 is tuff-dominated matrix-supported granule-to-pebble lithofacies (Tables 1, 2, 3 and 4). The lithofacies is also referred to as conglomeratic sandstone, and it has a high sandstone component and primarily consists of floating gravel. primarily grey, brown grey, and greenish-gray in hue, with a maximum thickness of 1 m, form A-3. The lithofacies, interbedded with thin pebbled sandstone, gritstone, or dark mudstone, develops massive structures, normal rhythms, reverse rhythms, weakly gravel orientations, and additional stratifications. And between pebbled sandstone, gritstone, or dark mudstone and conglomerate is a distinct erosional surface. Besides, the gritstone developed both trough cross-bedding and parallel bedding. The conglomerate in this lithofacies, mainly supported by sandstone matrix, has a small gravel grade, Poor to medium sorting, and varied rounding of subangular, sub-angular to sub-rounded, and rounded.

**Fig. 7:**
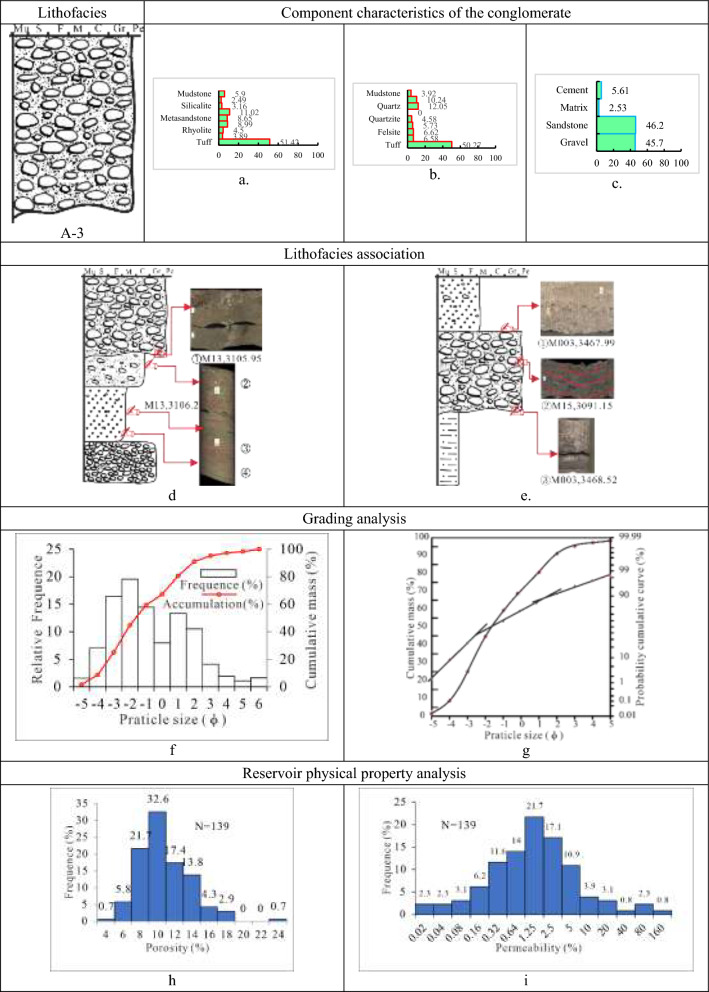
Parameter characteristics of the delta front conglomerate of class A-3 in the Baikouquan Formation (T_1_*b*), Northern Slope of Mahu Depression. (**a)** Gravel component (%), (**b)** Sandstone component (%); (**c)** Conglomerate composition (%); (**d)** ① Scour and fill structure, ② Weakly gravel orientations, ③ Parallel bedding in the sandstone, ④ Trough cross bedding; (**e)** ① Scour and fill structure, ② Normal rhythm in three phase, ③ Lithologic interface; (**f)** Grain distribution histogram and cumulative grading curve; (**g)** Cumulative probability curve; (**h)** Histogram of porosity distribution; (**i)** Permeability distribution histogram.

With an average gravel content of 45.7%, the lithofacies A-3 is dominated by volcaniclastic rocks (tuff), accounting for 51.4%, followed by magmatic rocks (granite and andesite) at 28.4%, sedimentary rocks (mudstone and diorite) and metamorphic rocks (metasandstone and quartzite), at low contents of 9.1% and 11.1%, respectively. The cement is calcite and ferriferous calcite, with a high average percentage of 5.6%, while the filler matrix is hydromica, kaolinite, chlorite, and mud, with a low average content of 2.5%. The A-3 lithofacies porosity and permeability histogram display an unimodal mode with a value of 8%-14% and (0.3–5) × 10^–3^ µm^2^, respectively. In comparison, the physical properties of Class A-3 conglomerates are superior to Class A-1 and Class A-2. An indication of the lithofacies A-3 is typically gravels floating on sand particles. With a grain size histogram showing a positive bimodal mode and a cumulative curve containing three parts (rolling, jumping, and suspending), this lithofacies’ deposit type is linked to the fan delta front detrital channel. These features reflect traits of traction current deposits under stable or moderate hydrodynamic conditions. (Fig. 7).

(4) Class A-4 is tuff-dominated gravel-supported granule-to-pebble lithofacies (Tables 1, 2, 3 and 4). Primarily brownish grey, greenish grey, purplish grey, and dark grey in hue, with a maximum thickness of 2 m, form A-4. The lithofacies, interbedded with thin pebbled sandstone, gritstone, or mudstone, develops massive structures, normal rhythms, graded beddings, reverse rhythms, weakly gravel orientations, scour and fill structures, and additional stratifications. And between different lithologies is a distinct erosional surface. The conglomerate in this lithofacies, mainly supported by gravel of each other, has a fine to medium gravel grade, medium to poor sorting, and moderately worse rounding of sub-angular and sub-angular to sub-rounded.

**Fig. 8:**
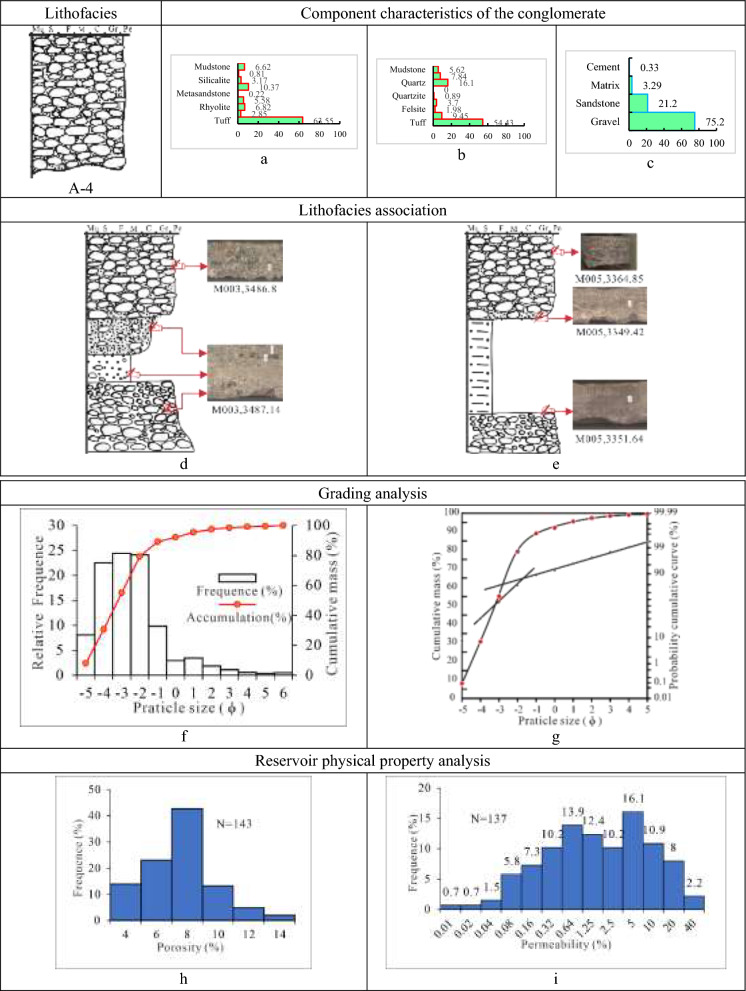
Parameter characteristics of the delta front conglomerate of class A-4 in the Baikouquan Formation (T_1_*b*), Northern Slope of Mahu Depression. (**a)** Gravel component (%), (**b)** Sandstone component (%); (**c)** Conglomerate composition (%); (**d**) The conglomerate is interlayered with thin pebbly sandstone, Lithologic gradient between conglomerate and sandstone, (**e)** The conglomerate is interlayered with thin brown pebbly siltstone, gravel supports the conglomerate, with weakly gravel orientations developed; (**f)** Grain distribution histogram and cumulative grading curve; (**g)** Cumulative probability curve; (**h)** Histogram of porosity distribution; (**i)** Permeability distribution histogram.

With an average gravel content of 75.2%, the lithofacies A-4 is dominated by volcaniclastic rocks (tuff), accounting for 63.6%, followed by magmatic rocks (granite and andesite) at 25.6%, sedimentary rocks (mudstone and siliceous rock) and metamorphic rocks (metasandstone and quartzite) at low contents of 9.8% and 1.1%, respectively. In contrast to A-3 lithofacies, A-4 lithofacies have virtually no metamorphic rock component at all. The cement is calcite and ferriferous calcite, with a low average percentage of 0.3%, while the filler matrix is hydromica, kaolinite, chlorite, and mudstone, with relatively a low average content of 3.3%. The A-4 lithofacies porosity histogram displays an unimodal mode with a value of 6%-8%. In contrast, the permeability histogram shows a low bimodal mode with a wide range of variation, with a value of (0.1–200) × 10^–3^ µm^2^. With a grain size histogram showing a high unimodal mode and a cumulative curve containing two parts (rolling and jumping, dominated by rolling), the lithofacies’ deposit type is linked to the fan delta front branch channel. (Fig. 8).

#### Fan delta plain conglomerate

1. Class B-1 is tuff and magmatic rocks-dominated gravel-supported granule-to-pebble lithofacies (Tables 5, 6, 7 and 8). Thick, massive rock, primarily amaranth, and brownness in hue, with a maximum thickness of 4 m, form B-1. The lithofacies, interbedded with thin brownish mudstone with a thickness of 10 cm to 30 cm, develops massive structures, weakly gravel orientations, scour and fill structure, and additional stratifications. And between different lithologies is a distinct mutational surface. The conglomerate in this lithofacies, mainly supported by gravel of each other, has a fine to medium gravel grade, medium to poor sorting, and moderately worse rounding of sub-angular and sub-angular to sub-rounded.

**Table 5:** Overall characteristics of the fan delta plain conglomerate of Baikouquan Formation (T_1_*b*), Northern Slope of Mahu Depression.

Litho	Well	Depth interval (m)	Rock type	Matrix	Cement	Pyroclastics	Mag	Met	Sediment
B-1	M101	3792.35–3798.11	Gr.-Pe	Lower	Low	Tuff	G	–	Mu
	M005	3430–3433.4	Gr.-Pe	Lower	Low	Tuff	G	–	–
	M11	3565.64–3567.1	Gr.-Pe	Lower	Low	Tuff	–	–	Mu
B-2	M009	3608–3613.7	Pe.-Co	Lower	Low	Tuff	–	–	Mu
	M009	3636.8–3640.05	Pe.-Co	Lower	Low	Tuff	–	–	Mu
	M003	3499.6–3502.8	Pe.-Co	Lower	Lower	Tuff	–	–	Mu
	M003	3546.4–3553.7	Co	Lower	Lower	Tuff	–	–	Mu
	M003	3578.6–3581.6	Pe.-Co	Lower	Lower	Tuff	–	–	Mu
B-3	M002	3480.7–3485.32	Co.-Bo	Lower	Low	Tuff	–	Q	–
	M002	3500.3–3506	Co.-Bo	Lower	Low	Tuff	F., A., G	Me	–
	M002	3526.5–3533	Pe.-Co	Lower	Low	Tuff	F	–	–
	M001	3469–3477.99	Pe.-Co	Lower	Low	Tuff	R., A	–	–
	M001	3486–3491.1	Pe.-Co	Lower	Low	Tuff	R., G	–	–
	M001	3510.04–3515.6	Co.-Bo	Lower	Low	Tuff	–	–	–
	M132	3268.09–3283.01	Co	Lower	Low	Tuff	–	–	–
B-4	M006	3410.97–3421.9	Pe.-Co	Higher	Lower	Tuff	G	–	Mu
	M006	3410.97–3421.9	Pe.-Co	Higher	Lower	Tuff	G	–	Mu
	M2	3463.8–3465.7	Pe.-Co	High	Low	Tuff	–	–	–
	X92	2504.71–2512.96	Pe.-Co	Higher	Lower	Tuff	R., A	Me	–
	X81	2552–2555.9	Pe.-Co	Higher	Lower	Tuff	F., G	–	–

Where Co., Pe., Gr., Bo. represent Cobble, Pebble, Granule, Boulder, respectively; F. A., R., G. represent Felsite, Andesite, Rhyolite, Granite, respectively; Me., Q. represent Metasandstone, Quartzite, respectively. Mu. and S. represent Mudstone and Silicalite, respectively.

**Table 6:** Structure feature of the fan delta plain conglomerate of Baikouquan Formation (T1*b*), Northern Slope of Mahu Depression.

Litho	Well	Supporting	Sorting	Structure	Petrographic color	Rounding
B-1	M101	Ss	Worse-Medium	Ms., Gwo., Sf	Fuchsia	Sa. to Sr
	M005	Ss	Bad	Ms	Taupe	Sa
	M11	Ss	Medium	Ms	Taupe gray, Maroon	Sa. to Sr
B-2	M009	Gs	Bad	Ms., Nr	Taupe gray	Sa. to Sr
	M009	Gs	Worse	Ms., Rr	Maroon	Sa., Sa. to Sr
	M003	Gs	Worse	Ms	Gray-brown, Taupe	Sa. to Sr
	M003	Gs	Bad	Ms., Go	Taupe	Sa., Sa. to Sr
	M003	Gs	Bad	Ms	Reseda, Taupe	Sa. to Sr
B-3	M002	Gs	Bad	Ms	Taupe	Sr
	M002	Gs	Bad	Ms	Taupe, Maroon	Sr
	M002	Gs	Bad	Ms	Taupe, Maroon	Sa. to Sr., Sr
	M001	Gs	Bad	Ms	Gray-brown, Taupe	Sa. to Sr., Sr
	M001	Gs	Bad	Ms., Rr	gray-brown, Mottle	Sr
	M001	Gs	Bad	Ms., Rr., Gwo	Gray-brown, Greenish brown	Sa., Sr
	M132	Gs	Worse	Gwo., Nr., Rr., Sf	Taupe	Sa. to Sr., Sr
B-4	M006	Ms	Bad	Ms	Taupe	Sa., Sr
	M006	Ms	Bad	Ms	Taupe	Sa., Sr
	M2	Ms	Bad	Ms	Maroon, Taupe, Dark brown	Sa., Sr
	X92	Ms	Bad	Ms	Grey	Sr
	X81	Ms	Bad	Ms	Taupe, Mottle	Sr

Where GS., Ms., Ss. represent Gravel-Supported, Mudstone-Supported, Sandstone-Supported, respectively; Ms. Gwo., Nr., Rr., Sf., Go. represent Massive structure, Gravel weakly-oriented, Normal rhythm, Reverse rhythm, Scour and fill, Gravel oriented, respectively; Sa., Sr. represent Sub-angular, Sub-rounded, respectively.

**Table 7:** Component analysis of the fan delta plain conglomerate of Baikouquan Formation (T_1_*b*), Northern Slope of Mahu Depression.

Litho	Well	Overall	Tuff/%	F./%	R.%	A./%	Me./%	G./%	S./%	Q./%	Mu./%
B-1	M101	73.8	35.4	0	0	0	0	27.4	0	0	11
	M005	72	50	0	0	0	0	22	0	0	0
	M11	87	80.5	0	0	0	0	0	0	0	6.5
B-2	M009	82	57	0	0	3	0	0	0	5	17
	M009	82.5	71	0	0	2.5	0	0	0	0	9
	M003	79	64	0	0	0	5	0	0	0	10
	M003	67.33	47.67	0	0	0	0	0	0	0	19.67
	M003	71	37	0	20	0	0	0	0	0	14
B-3	M002	80.5	59	2	3	1.5	0	1.5	0	13.5	0
	M002	76.2	39.2	10.6	2.4	6.8	11.4	5	0.4	0	0.4
	M002	60.67	48.67	7.33	3.33	0.33	0.67	0	0	0	0.33
	M001	83	55.33	0	12.5	7.33	0	3.83	0	1.67	2.5
	M001	78.25	56.75	1.75	9	1.25	0	9.5	0	0	0
	M001	50.2	45.4	0	1.4	2	0	1.4	0	0	0
	M132	76.67	70.67	1.67	0	0	0.67	2.67	0	0.67	0
B-4	M006	62	45.6	0	0	0	0	8.2	0	0	8.2
	M006	62	45.6	0	0	0	0	8.2	0	0	8.2
	M2	48.25	39.5	0	1.5	0	1.25	1	1.25	0	3.75
	X92	67.5	32.5	5	0	6	12.5	0	0	1.5	10
	X81	45.5	22	11.5	0	2.5	0	9.5	0	0	0

**Table 8:** Fill characteristics of the fan delta plain conglomerate of Baikouquan Formation (T_1_*b*), Northern Slope of Mahu Depression.

Litho	Well	Matrix/%	Matrix component	Cement/%	Cement component
B-1	M101	2.6	Iron stains mud, Magnetite, Kaolinite, Biotite	0.4	Magnetite
	M005	4	Mud, Hydrobiotite, Kaolinite	–	–
	M11	1.5	Kaolinite, Hydrobiotite	1.5	Limonite
B-2	M009	3	Iron stains mud	–	–
	M009	2.5	Iron stains mud, Mud	–	–
	M003	2	Iron stains mud	2.5	Iron
	M003	1	Iron stains mud	2.33	Iron stains mud, Iron
	M003	3.5	Iron stains mud, Hydromica, Mud	0.5	Iron
B-3	M002	3	Kaolinite, Mud	–	–
	M002	2.4	Mud	–	–
	M002	2.67	Mud, Hydromuscovite	–	–
	M001	3	Mud	0.5	Calcite
	M001	2.25	Mud	–	–
	M001	4.4	Iron stains mud, Mud	–	–
	M132	1.67	Iron stains mud, Mud	–	–
B-4	M006	12	Hydromica mud, Hydromica, Kaolinite, Chlorite	2.2	–
	M006	12	Hydromica mud, Hydromica, Kaolinite, Chlorite	2.2	–
	M2	14.25	Iron oxide stains mud, Iron stains mud, Mud, Kaolinite	0	–
	X92	9.5	Mud	1.5	Calcite
	X81	8.5	Mud	1.5	Calcite, Analcite

**Fig. 9:**
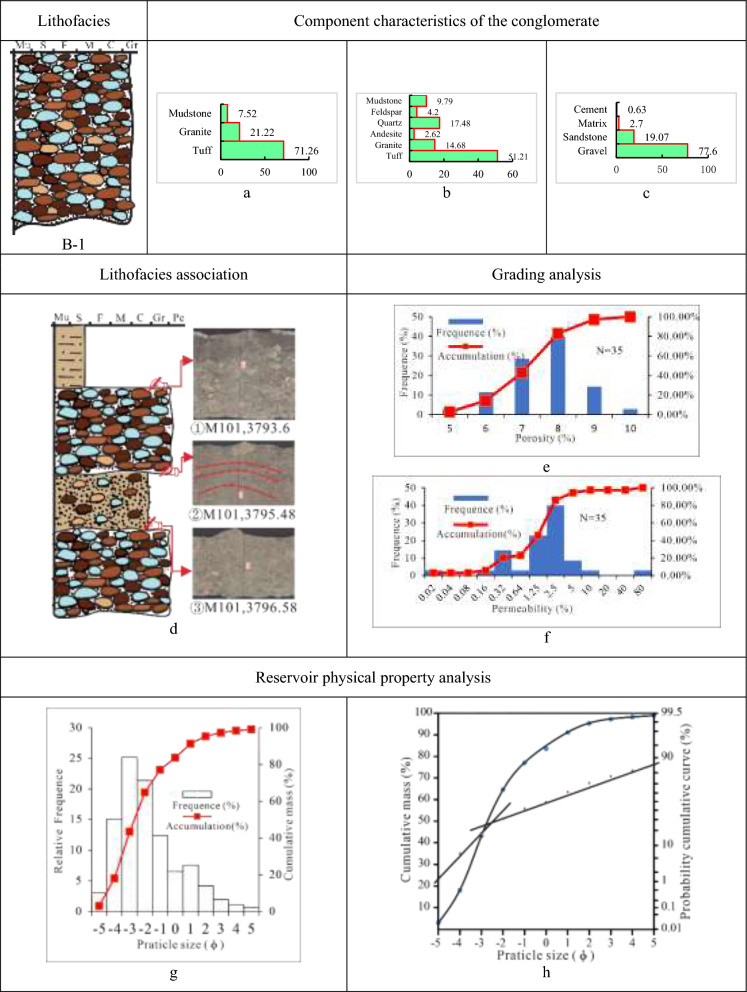
Parameter characteristics of the delta plain conglomerate of class A-1 in the Baikouquan. (**a)** Gravel component (%), (**b)** Sandstone component (%); (**c)** Conglomerate composition (%); (**d)** ① Discontinuity surface between conglomerate and mudstone, ② Weakly gravel orientations, ③ Normal rhythm, and the bottom develop scour and filling structure; (**e)** Histogram of porosity distribution; (**f)** Permeability distribution histogram; (**g)** Grain distribution histogram and cumulative grading curve; (**h)** Cumulative probability curve.

With an average gravel content of 77.6%, the lithofacies B-1 is dominated by volcaniclastic rocks (tuff), accounting for 71.3%, followed by magmatic rocks (granite) at 21.2%, sedimentary rocks (mudstone) at a low content of 7.5%. The cement is magnetite, with a low average percentage of 0.6%, and the filler matrix is biotite, kaolinite, chlorite, and argillaceous covered with iron, with a low average content of 2.7%. The B-1 lithofacies porosity histogram displays a low unimodal mode with a value of 6%-9%, whereas the permeability histogram shows a high unimodal mode, with a value of (0.3–2.5) × 10^–3^ µm^2^. Therefore, the reservoir of B-1 has a slightly high permeability but a low porosity. With a grain size histogram showing a high unimodal mode and a cumulative curve containing two parts (rolling and jumping, while dominated by rolling), the lithofacies’ deposit type is linked to the fan delta plain braided channel (Fig. 9).

(2) Class B-2 is tuff and sedimentary rocks-dominated gravel-supported pebble-to-cobble lithofacies (Tables 5, 6, 7 and 8). Thick, massive rock, primarily brownness, maroon, taupe, and brown-grey in hue, with a maximum thickness of 6 m, form B-2. The lithofacies, interbedded with dark medium to coarse sandstone and pebbled mudstone with a thickness of 20 cm to 50 cm, develops massive structures, normal rhythms, reverse rhythms, gravel orientations, and additional stratifications. And between different lithologies is a scour and fill structure or distinct mutational surface. The conglomerate in this lithofacies, mainly supported by gravel, has a medium to coarse gravel grade, poor sorting, and moderately worse rounding of sub-angular and sub-angular to sub-rounded.

**Fig. 10:**
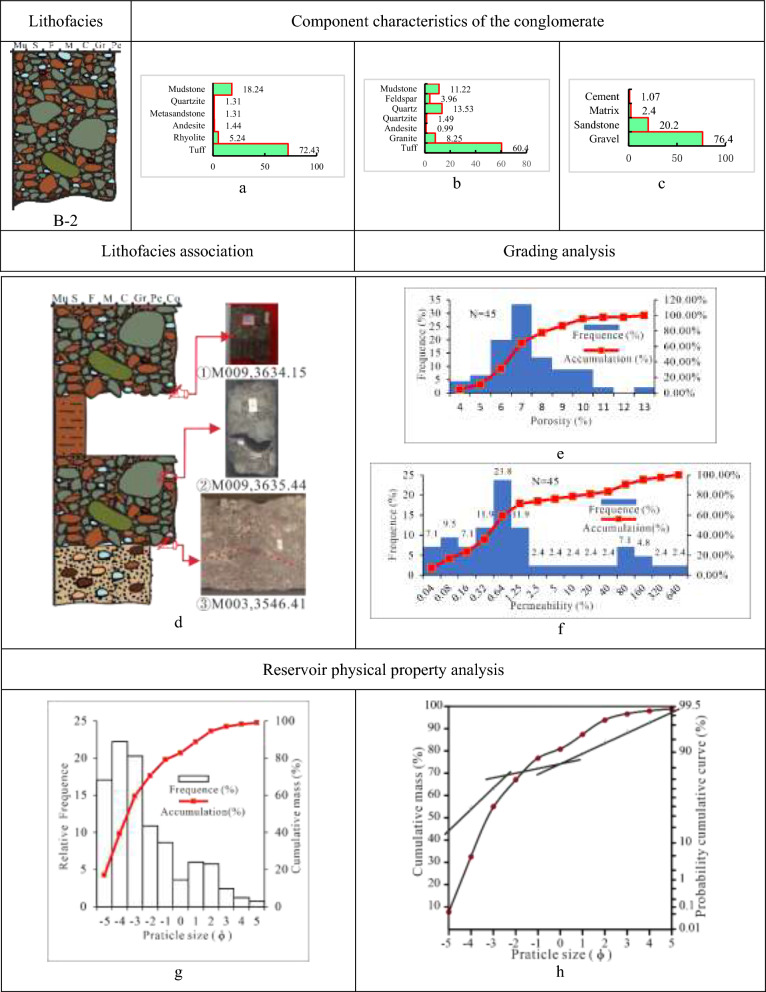
Parameter characteristics of the delta plain conglomerate of class A-2 in the Baikouquan. (**a)** Gravel component (%), (**b)** Sandstone component (%); (**c)** Conglomerate composition (%); (**d)** ① Discontinuity surface between conglomerate and mudstone, ② Gravel-supported cobble, the gravel floats in the conglomerate, and has poor sorting with moderate rounding, ③ Reverse rhythm, with the gravel having a weak orientation in pebbly sandstone; (**e)** Histogram of porosity distribution; (**f)** Permeability distribution histogram; (**g)** Grain distribution histogram and cumulative grading curve; (**h)** Cumulative probability curve.

With an average gravel content of 76.4%, the lithofacies B-2 is dominated by volcaniclastic rocks (tuff), accounting for 72.4%, followed by sedimentary rocks (mudstone) at 18.2% and magmatic rocks (rhyolite) at a low content of 5.1%. The filler matrix is made up of hydromica, mud, and mud covered in iron, with an average content of 2.4%, and the cement is argillaceous cementation, with a low average proportion of 1.1%. The porosity and permeability histograms of the B-2 lithofacies show a high unimodal mode of 6%–8% and (0.04–1.25) × 10^–3^ µm^2^, respectively. As a result, there is limited porosity and permeability in the B-2 reservoir. The deposit type of the lithofacies is associated with the fan delta plain detrital channel according to a grain size histogram that displays a high multimodality mode and a cumulative curve that displays a low slope (Fig. 10).

(3) Class B-3 is tuff-dominated gravel-supported cobble-to-boulder lithofacies (Tables 5, 6, 7 and 8). Thick, massive rock with a maximum thickness of 10 m and a typical color of brown, taupe, brick red, and olive drab characterize Class B-3. The lithofacies, interbedded with thin dark pebbled gritstone with a thickness of 20 cm to 50 cm and dark mudstone with a thickness exceeding 100 cm, develops massive structures, normal rhythms, reverse rhythms, weakly gravel orientations, and additional stratifications. Between different lithologies is a scour and fill structure or distinct mutational surface. The conglomerate in this lithofacies, mainly supported by gravel, has a cobble to boulder gravel size, extremely poor sorting, and moderate rounding of sub-angular to sub-rounded and sub-rounder.

**Fig. 11:**
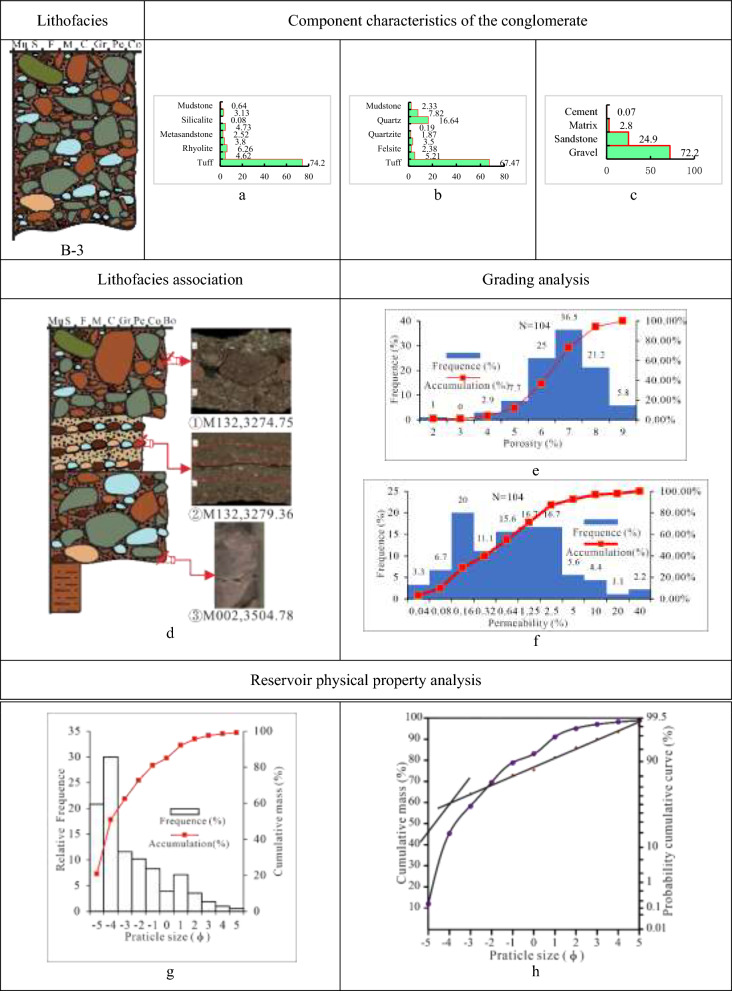
Parameter characteristics of the delta plain conglomerate of class A-3 in the Baikouquan. (**a)** Gravel component (%), (**b**) Sandstone component (%); (**c)** Conglomerate composition (%); (**d**) ① Boulder, ② The gravel has a weak orientation in pebbly sandstone, and fill and scour structure has formed at the bottom, ③ Discontinuity surface between conglomerate and mudstone, ④ Reverse rhythm, with the gravel having a weak orientation in pebbly sandstone; (**e**) Histogram of porosity distribution; (**f**) Permeability distribution histogram; (**g)** Grain distribution histogram and cumulative grading curve; (**h)** Cumulative probability curve.

With an average gravel content of 72.2%, the lithofacies B-3 is dominated by volcaniclastic rocks (tuff), accounting for 74.2%, followed by magmatic rocks (felsite, rhyolite, andesite, and granite) at 19.41%, metamorphic rocks (metasandstone and quartzite) at a low content of 5.1%, and no sedimentary rocks. The filler matrix is made up of hydromuscovite, kaolinite, mud, and mud covered in iron, with an average content of 2.8%, and the cement is calcite, with an extremely low average proportion of 0.07%. The porosity histogram of the B-3 lithofacies shows an unimodal mode of 6%–8% and the permeability histogram shows a dwarf unimodal mode of (0.04–2.5) × 10^–3^ µm^2^. As a result, there is a limited porosity and slightly higher permeability in the B-3 reservoir. The deposit type of the lithofacies is associated with the fan delta plain detrital channel according to a grain size histogram that displays a high unimodality mode and a cumulative curve that displays a low slope (Fig. 11).

(4) Class B-4 is tuff, magmatic rocks and sedimentary rocks-dominated matrix-supported pebble-to-cobble lithofacies (Tables 5, 6, 7 and 8). Thick, massive rock with a maximum thickness of 5 m and a typical color of brown, maroon, and brown-grey characterize Class B-4. The lithofacies, interbedded with thin dark pebbled mudstone with a thickness of 10 cm to 30 cm, develops massive structures and graded beddings. Between different lithologies is a scour and fill structure or distinct mutational surface. The conglomerate in this lithofacies, which is mainly supported by matrix, has a medium to coarse gravel grade, extremely poor sorting, and a broad rounding of sub-angular, sub-angular to sub-rounded, and sub-rounded.

**Fig. 12:**
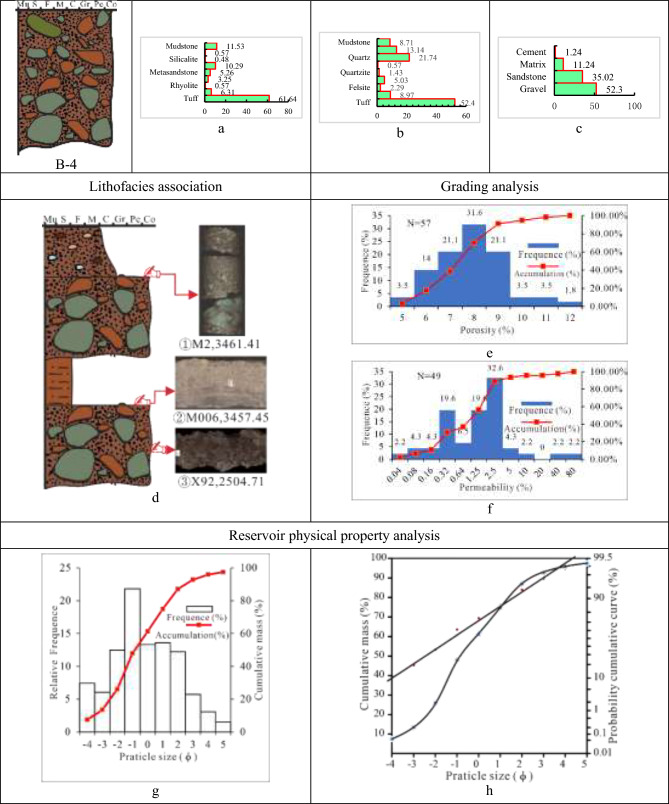
Parameter characteristics of the delta plain conglomerate of class A-4 in the Baikouquan. (**a)** Gravel component (%), (**b**) Sandstone component (%); (**c**) Conglomerate composition (%); (**d**) ① Normal rhythm, the upper part is pebbly sandstone, and the lower part is matrix-supported conglomerate, ② Lithologic gradient between conglomerate and sandstone, ③ Debris flow, matrix-supported conglomerate; (**e)** Histogram of porosity distribution; (**f**) Permeability distribution histogram; (**g)** Grain distribution histogram and cumulative grading curve; (**h**) Cumulative probability curve.

With an average gravel content of 52.3%, the lithofacies B-4 is dominated by volcaniclastic rocks (tuff), accounting for 61.64%, followed by magmatic rocks (felsite and granite) at 20.42% and sedimentary rocks (mudstone) at a low content of 6.28%. The filler matrix is composed of hydromica, hydromica mud, kaolinite, chlorite, and mud covered in ferric oxide, with a high average content of 11.4%, and the cement is calcite and zeolite, with a low average proportion of 1.24%. The porosity histogram of the B-4 lithofacies shows a high unimodal mode of 6%–9% and the permeability histogram shows a high bimodal mode of (0.32–0.64 and 1.25–5) × 10^–3^ µm^2^. As a result, there is a limited porosity and slightly higher permeability in the B-4 reservoir. The deposit type of the lithofacies is associated with the fan delta plain braided channel according to a grain size histogram that displays a low unimodality mode and a cumulative curve with no obvious mutational point (Fig. 12).

The new classification system introduced in this study overcomes the limitations of traditional methods by integrating both geological and engineering factors. This hybrid approach ensures more accurate reservoir characterization, particularly in the context of hydraulic fracturing, by considering variations in gravel composition, cementation, and support type. By classifying the conglomerates into distinct types based on these criteria, the proposed system provides a robust framework for optimizing the exploration and development of the T1b conglomerate reservoirs in the Mahu Depression.

## Discussion

### Comparison of Lithofacies’ characteristics

The primary distinctions among the various lithofacies of the conglomerate are evident in gravel size, supporting type, gravel composition, and cementation. The conglomerates from the fan delta front (Class A) and fan delta plain (Class B) demonstrate significant variations in their physical properties, which influence both their reservoir quality and hydraulic fracturing potential.

Class A-1 is predominantly composed of volcaniclastic rocks (tuff), metamorphic rocks (quartzite and metasandstone), and magmatic rocks (felsite and andesite), with gravel sizes ranging from cobble to boulder. This lithofacies is mainly gravel-supported and exhibits high average cement content and low filler matrix content. Class A-2 is similar in composition but is matrix-supported, with a pebble-to-cobble gravel size. It shows lower cement content and higher filler matrix content compared to A-1. Class A-3 is predominantly composed of tuff, with a granule-to-pebble gravel size and sandstone matrix support. It has a high average cement content, low filler matrix content, and superior physical properties. Finally, Class A-4, also primarily composed of tuff, exhibits a gravel-supported granule-to-pebble size with low average cement and filler matrix content. In terms of reservoir quality, Class A-3 shows the best physical properties, followed by A-4, A-1, and A-2, respectively. Class A-1 and A-4 belong to the fan delta front main channel deposits, A-2 is from the fan delta front branch channel deposits, and A-3 originates from the fan delta front clastic channel deposits (Table 9).

**Table 9:** An overview of the traits of those different lithofacies.

Facies	Litho-facies	Gravel size	Supporting types	Component	Cementation	Poroperm	Cumulative size distribution curve
A	A-1	Cobble-to-boulder	Gravel-supported	Tuff, quartzite, metasandstone, felsite, and andesite	Strong	Poor	Rolling and jumping
	A-2	Pebble-to-cobble	Matrix-supported (mudstone)	Tuff, felsite, and andesite	Weak	Poor	Low slope and no evident node
	A-3	Granule-to-pebble	Matrix-supported (sandstone)	Tuff	Strong	Good	Rolling, jumping, and suspending
	A-4	Granule-to-pebble	Gravel-supported	Tuff	Weak	Better	Rolling
B	B-1	Granule-to-pebble	Gravel-supported	Tuff and granite	Weak	Worse	Rolling and jumping, dominated by rolling
	B-2	Pebble-to-cobble	Gravel-supported	Tuff and mudstone	Weak	Worse	Low slope
	B-3	Cobble-to-boulder	Gravel-supported	Tuff	Weak	Worse	Low slope
	B-4	Pebble-to-cobble	Matrix-supported (sandstone)	Tuff	Weak	Poor	No obvious mutational point

Among the fan delta plain conglomerates, Class B-1 is dominated by volcaniclastic (tuff) and magmatic rocks (granite), gravel-supported, with a granule-to-pebble size and low cement and filler matrix content. Class B-2 is primarily composed of tuff and sedimentary rocks (mudstone), gravel-supported with a pebble-to-cobble size, also with low cement and filler matrix content. Class B-3, primarily composed of tuff, is gravel-supported and has a cobble-to-boulder gravel size, with low cement and filler matrix content. Class B-4, composed of tuff, magmatic rocks, and sedimentary rocks, is matrix-supported with a pebble-to-cobble gravel size and a low average cement content, but with a high average filler matrix content. Reservoir quality in the fan delta plain conglomerates is generally lower than in the fan delta front conglomerates, with Class B-1 showing the best physical properties, followed by Class B-4, Class B-3, and Class B-2. Class B-1 is associated with fan delta plain braided channel deposits, while Class B-2, B-3, and B-4 are linked to fan delta plain detrital channel deposits (Table 9).

### Hydrofracturing evaluation of the conglomerate and the most favorable lithofacies

Research has shown that the gravel structure significantly affects hydraulic fracture propagation. Coarse gravel size, poor sorting, and high gravel content tend to inhibit the formation of complex fracture networks, resulting in less effective fracture propagation^9^. Class A-3 has the best physical properties for hydraulic fracturing due to its fine to medium gravel size, floating gravel within a sandstone matrix, low matrix content, and high cement content. These characteristics facilitate complex fracture networks, thus optimizing oil and gas production. The A-3 lithofacies’ grading curve, which includes rolling, jumping, and suspending, further supports its suitability for hydraulic fracturing. From an oil and gas exploration perspective, A-3 represents the most favorable lithofacies for creating effective hydraulic fractures.

**Fig. 13:**
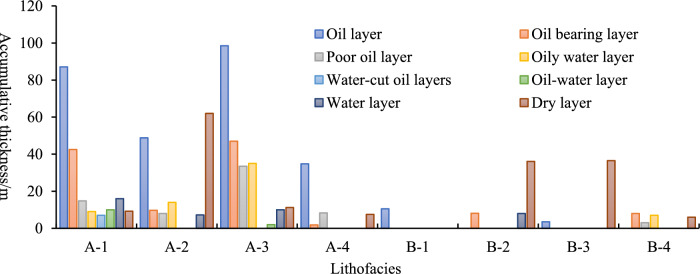
Cumulative thickness histogram spanning multiple lithofacies’ oil test data.

Oil layer testing data (Fig. 13) further supports these conclusions. Class A (fan delta front conglomerates) generally exhibits better oil layer testing results, with a significant accumulated thickness of oil-bearing deposits. In contrast, Class B (fan delta plain conglomerates) shows poor oil layer testing results, particularly in the tight zone and water zones. Among the Class A lithofacies, A-3 stands out as the best performer in oil layer testing, with a cumulative thickness of oil-bearing deposits exceeding 100 m. This reinforces the argument that A-3 is the most favorable lithofacies for both hydraulic fracturing and oil production.

### Reservoir intervals and their implications for hydrocarbon exploration

The T_1_*b* conglomerate reservoirs in the North Slope of the Mahu Depression exhibit significant spatial heterogeneity due to variations in depositional environment, lithology, and cementation. The fan delta front conglomerates, particularly Class A-3, are associated with the most productive reservoir intervals, showing better physical properties, higher porosity, and permeability, as well as the potential for more effective hydraulic fracturing. These intervals are characterized by more stable grain sorting, better fracturing potential, and a higher proportion of favorable lithofacies, making them prime targets for hydrocarbon exploration. On the other hand, fan delta plain conglomerates (Class B) generally display poorer reservoir quality, with lower porosity and permeability. The reservoirs in these intervals tend to be more challenging for hydraulic fracturing due to the less favorable grain size, poorer sorting, and higher matrix content. Although Class B-1 shows relatively better reservoir properties compared to other fan delta plain lithofacies, its reservoir quality is still inferior to the fan delta front conglomerates.

The distinction between fan delta front and fan delta plain conglomerates has important implications for exploration and development strategies. Fan delta front reservoirs, especially Class A-3, are ideal for targeted hydraulic fracturing and oil production due to their superior physical properties, while fan delta plain reservoirs may require more careful evaluation and advanced fracturing techniques to achieve economically viable production.

### Limitations and areas for future research

While this study provides valuable insights into the classification of conglomerate reservoirs in the T_1_*b* Formation, there are some limitations. One of the key challenges lies in the complex and variable nature of conglomerate deposits, which may not be fully captured by the current classification system. Further research is needed to refine the classification system, particularly in terms of integrating more detailed reservoir simulation models and advanced fracture propagation simulations that can better account for variations in the subsurface conditions.

Moreover, additional core data, well log analysis, and field testing will be essential to validate the proposed classification system and its effectiveness in predicting reservoir performance and hydraulic fracturing outcomes. Future studies should also explore the impact of other geological factors, such as mineralogy, diagenesis, and structural features, on fracture propagation and fluid flow in conglomerate reservoirs.

## Conclusions

This study addresses the challenges in selecting target intervals for hydraulic fracturing in the highly heterogeneous conglomerate reservoirs of the T_1_*b* Formation, North Slope of the Mahu Depression. By integrating geological and engineering perspectives, a novel classification system fo2r conglomerate lithofacies has been developed to identify favorable reservoir intervals. Key conclusions are as follows:

1. The conglomerates of the T_1_*b* Formation were categorized into two main groups—fan delta front and fan delta plain conglomerates—each further divided into four distinct types based on factors such as gravel size, support type, cementation degree, and composition. This classification scheme enhances the understanding of the depositional environment and the impact of lithological properties on reservoir quality.
2. The fan delta front conglomerates (Class A) generally exhibit superior reservoir quality compared to fan delta plain conglomerates (Class B). Among the fan delta front lithofacies, Class A-3 (tuff-dominated, matrix-supported granule-to-pebble lithofacies) stands out with the best physical properties, such as higher porosity and permeability, due to its fine-to-medium gravel size, high cement content, and low matrix content. These characteristics support effective fracture propagation and complex fracture networks, which are essential for enhancing hydrocarbon production.
3. Class A-3 lithofacies is the most favorable target for hydraulic fracturing, due to its geological and mechanical properties that promote complex fracture networks, enhancing permeability and fluid flow. Oil layer testing further confirms its potential, showing significant oil-bearing thickness. Pore networks in fan delta front conglomerates, especially Class A-3, are well-developed, improving fluid connectivity, while fan delta plain conglomerates have more limited pore networks, resulting in lower permeability and reservoir quality. These findings emphasize the importance of lithofacies-based classification for optimizing hydraulic fracturing.
4. The new classification system provides a robust framework for identifying and optimizing hydraulic fracturing intervals in heterogeneous conglomerate reservoirs. By integrating geological attributes with engineering considerations, this approach offers a valuable tool for future hydrocarbon exploration and development in conglomerate reservoirs similar to those found in the Mahu Depression.

In summary, this research offers a comprehensive classification of conglomerate lithofacies in the T_1_*b* Formation, emphasizing Class A-3 as the most promising lithofacies for hydraulic fracturing. The insights gained here are expected to guide the selection of optimal fracturing intervals and improve production strategies in similar complex reservoirs. Future studies should further validate this classification with additional field data and explore the influence of diagenesis and structural features on reservoir performance.

## Data Availability

The data that support the findings of this study are available from the Xinjiang Oil Field of PetroChina, but restrictions apply to their public availability as they were used under license for the current study. Data may be made available to researchers upon reasonable request and with explicit permission from the Xinjiang Oil Field of PetroChina. To initiate a data request, please contact the first author Dr. Xiangyang Li, directly at lxy.cup@outlook.com.
